# Effect of In Vitro Gastrointestinal Digestion on the Polyphenol Bioaccessibility and Bioavailability of Processed Sorghum (*Sorghum bicolor* L. Moench)

**DOI:** 10.3390/molecules29225229

**Published:** 2024-11-05

**Authors:** Aduba Collins, Nidhish Francis, Kenneth Chinkwo, Abishek Bommannan Santhakumar, Christopher Blanchard

**Affiliations:** 1 School of Dentistry and Medical Sciences, Charles Sturt University, Wagga Wagga, NSW 2650, Australia; njok@csu.edu.au (A.C.); kchinkwo@csu.edu.au (K.C.); asanthakumar@csu.edu.au (A.B.S.); cblanchard@csu.edu.au (C.B.); 2 School of Agricultural, Environmental and Veterinary Sciences, Charles Sturt University, Wagga Wagga, NSW 2650, Australia; 3 Gulbali Institute, Charles Sturt University, Wagga Wagga, NSW 2650, Australia

**Keywords:** pigmented sorghum, bioaccessibility, Caco-2 intestinal transport, simulated digestion

## Abstract

Sorghum is a significant source of polyphenols, whose content, antioxidant properties and bioaccessibility may be modulated by digestion. Studies have reported sorghum polyphenol changes after simulated digestion. However, the effects of simulated digestion on processed, pigmented sorghum are unknown. This study investigated the bioaccessibility and bioavailability of black (BlackSs and BlackSb), red (RedBa_1_, RedBu_1_, RedBa_2_, RedBu_2_) and white (WhiteLi_2_ and White Li_2_) sorghum samples using a Caco-2 in vitro model. Ultra high performance liquid chromatography—online 2,2′-azino-bis (3-ethylbenzothiazoline-6-sulfonic acid) (UHPLC–online ABTS)—and quadrupole time-of-flight liquid chromatography mass spectra (QTOF LC–MS) facilitated the identification of digested and transported compounds. Simulated digestion showed increased bioaccessibility and total phenolic content (TPC) for BlackSs by 2-fold. BlackSs and BlackSb exhibited high antioxidant capacities, with variations dependent on processing in other varieties. Kaempferol-3-*O*-xyloside exhibited a 4-fold increase in TPC following digestion of processed BlackSs and BlackSb but was absent in the others. BlackSs, BlackSb, and RedBu_1_ revealed twelve bioaccessible and Caco-2 transported compounds not previously reported in sorghum, including trans-pinostilbene, tryptophan and maackin a. This study demonstrates that in vitro digestion increases the bioaccessiblity of sorghum polyphenols through the process of cellular biotransformation, possibly improving transport and bioactivity in vivo.

## 1. Introduction

The growing recognition of the health benefits associated with whole grains has stimulated research into the bioaccessibility and bioavailability of sorghum (*Sorghum bicolor* L. Moench) following digestion [1,2]. Bioaccessibility describes the fraction of ingested compounds that are released from the food matrix, solubilized in the gastrointestinal tract and available for absorption into systemic circulation [3]. The amount that reaches the site of action intact is known as the bioavailability [4]. Wholegrain sorghum is renowned for its antioxidant and radical scavenging activity, primarily driven by its abundance in polyphenolic compounds; however, it is primarily used as livestock feed. Sorghum’s phytochemicals, including flavonoids and phenolic acids, contribute significantly to the overall antioxidant activity of sorghum [5]. Among its diverse varieties, darker pigmented sorghum has emerged as a promising candidate due to its potential health-promoting effects—including anti-diabetic, anti-cancer and anti-inflammatory activities—that are linked largely to its unique polyphenolic profile [6,7]. The antioxidative properties of sorghum not only enhance its shelf-life stability, but also offer potential health benefits by combating oxidative stress and associated chronic diseases [8,9].

A majority of wholegrains are not consumed in their raw form, but undergo domestic or industrial processing prior to consumption. Sorghum processing through cooking and fermentation is of significant interest due to its impact on the health benefits and bioavailability of the grain’s phenolic compounds [10]. Our previous study determined that sorghum fermentation and cooking processes increased the total phenolic content and antioxidant capacity, which resulted in highly detectable polyphenols released from the breakdown of cell-wall structures [11]. Several other processing technologies, such as extrusion and ultra-fine grinding, can enhance the bioaccessibility of polyphenols from wholegrain sorghum by introducing newly transformed compounds into the digestive matrix [4]. An investigation by Hole et al. (2013) [12] determined that extrusion increased the total bioaccessibility of oat and barley bound phenolic acids in weanling pigs by 29% and 14%, respectively. An improvement in the bioaccessibility of ferulic acids, *p*-coumaric and sinapic has also been reported in bran-enriched wheat bread using an in vitro gastrointestinal model [13]. However, the impact of gastrointestinal digestion of sorghum polyphenols for attributed health benefits is unknown. Digestion may influence the bioavailability of these processed sorghum compounds by understanding how cooking and fermentation affect their stability, solubility, and absorption during the digestive process. This approach allows for the evaluation of how these processing methods alter the release of bioactive compounds, providing insights into their potential health outcomes and guiding the optimization of processed sorghum for nutraceutical uses.

In vitro digestion models are convenient and simple tools used to replicate the enzymatic and environmental conditions of the gastric and intestinal phases, providing insights into how compounds are broken down and absorbed [2]. Despite the documented antioxidant and health-promoting properties of sorghum polyphenols, a critical gap exists in understanding their bioaccessibility. A small number of studies have assessed the bioaccessibility of raw or extruded sorghum polyphenols [2,14]. However, a complete study on the in vitro digestion of cooked and fermented black, red and white sorghum varieties has not been reported. Sorghum phenolic compounds need to resist digestive degradation for systemic absorption into the circulatory system and in vivo bioactivity. Therefore, it is vital to investigate their stability throughout the digestive process and their bioavailability post-absorption to fully understand their potential health benefits.

The colorectal cancer cell line, Caco-2, is a useful assay utilised to simulate intestinal absorption in vitro [15,16]. This cell line forms a monolayer with tight junctions, mimicking the intestinal barrier and allowing for the evaluation of the transport and permeability of compounds across the epithelial layer [16]. In vitro digestion and intestinal transport are critical factors influencing the physiological health outcomes associated with sorghum polyphenols. During in vitro digestion, polyphenols undergo chemical transformations and interactions with digestive enzymes that can affect their stability and bioavailability. As previously mentioned, the efficiency of intestinal transport mechanisms, including uptake by epithelial cells and passage into the systemic circulation following digestion, determines the extent to which these compounds can exert their beneficial effects. Enzymatic activity, micro-transporters, physiological pH fluctuations and optimum temperatures can influence the pharmacokinetics and level of absorption of polyphenols [17]. Therefore, determining sorghum phenolic bioaccessibility and bioavailability is crucial for assessing the potential health outcomes associated with dietary polyphenol consumption.

The primary objective of this study is to investigate the impact of simulated gastrointestinal digestion on the bioaccessibility and bioavailability of processed sorghum polyphenols. Also, this study aims to identify and characterize bioaccessible phenolic compounds that significantly contribute to sorghum’s antioxidant properties. In summary, this study addresses a significant gap in the current literature by exploring the bioaccessibility of sorghum polyphenols, thereby enhancing our understanding of their potential health benefits. The findings from this study may contribute to the development of dietary recommendations and functional food formulations aimed at optimising the delivery and efficacy of sorghum polyphenols for promoting human health.

## 2. Results

### 2.1. Effect of Simulated Gastrointestinal Digestion on the Total Phenolic Content (TPC) of Processed Sorghum

Raw undigested BlackSb demonstrated the highest phenolic content (6.05 ± 1.82 mg/g GAE) and BlackSs had the second highest TPC (5.05 ± 1.12 mg/g GAE) (Table 1). The TPC of RedBu_1_, RedBa_1_, RedBu_2_ and RedBa_2_ ranged from 0.47 ± 0.14 to 0.67 ± 0.08 mg/g GAE, while the phenolic contents of WhiteLi_1_ and WhiteLi_2_ were 0.52 ± 0.06 and 0.52 ± 0.05 mg/g GAE. Post-simulated digestion, the processed BlackSs exhibited an 82%, 83% and 96% increase in phenolic content for cooked, fermented and fermented-cooked samples, respectively.

Digested BlackSb also resulted in a rise in phenolic content by approximately 66%, 61% and 54% for cooked, fermented and fermented-cooked samples. There was a significant increase (*p <* 0.0001) in TPC post-digestion in fermented and fermented-cooked BlackSb and BlackSs; however, cooking had no significant difference (*p* > 0.05) in phenolic contents after digestion. Fermented RedBa_1_ exhibited with a 2-fold increase in total phenolic content after digestion, followed by an increase of 83%, 57%, and 54% for RedBu_1_, RedBu_2_ and RedBa_2_, respectively. Interestingly, total phenolic content was the highest after RedBu_2_ was cooked and digested. Post-digestion, the phenolic content of the white pericarp varieties significantly increased (*p <* 0.0001) with the highest TPC (83%) detected after fermentation of WhiteLi_1_ and 71% after cooking of WhiteLi_2_.

### 2.2. Effect of Simulated Digestion on the Antioxidant and Radical Scavenging Activity of Processed Sorghum

**Figure 1:**
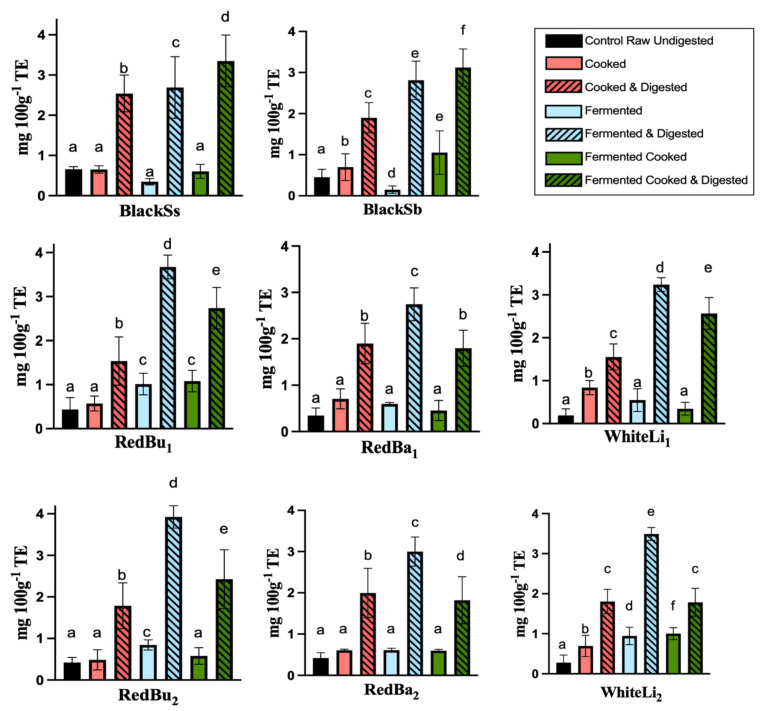
Changes in the total ABTS antioxidant activity of processed sorghum phenolic extracts before and after simulated gastrointestinal digestion. Data are expressed as mg 100^−1^ g TE, and represent mean ± SD; *n =* 9. Significant differences within and between sorghum samples are represented by different letters. R, raw; C, cooked; F, fermented; FC, fermented and cooked; WhiteLi_1_, Liberty from Bellata; WhiteLi_2_, Liberty from Croppa Creek; RedBa_1_, Bazley from Bellata; RedBa_2_, Bazley from Croppa Creek; RedBu_1_, Buster from Bellata; RedBu_2_, Buster from Croppa Creek; BlackSs, Shawaya short black 1; BlackSb, Shawaya black.

The ABTS and FRAP assays were utilised to evaluate the collective antioxidant capacity and radical scavenging properties in coloured and colourless sorghum varieties. Digested samples of processed sorghum wholegrains demonstrated a significant increase (*p* < 0.05) in ABTS for all eight sorghum varieties (Figure 1). Prior to digestion, BlackSs exhibited the highest antioxidant activity (0.66 ± 0.06 mg 100 g^−1^ TE), followed by BlackSb (0.45 ± 0.19 mg 100 g^−1^ TE), RedBu_1_ (0.44 ± 0.27 mg 100 g^−1^) and RedBa_2_ (0.42 ± 0.13 mg 100 g^−1^ TE). Digested BlackSb exhibited a 6-fold increase (0.45 ± 0.19 to 3.12 ± 0.46 mg/100 g^−1^ TE) compared to the control, whereas BlackSs increased 5-fold from 0.66 ± 0.06 to 3.35 ± 0.64 mg 100^−1^ TE for the fermented-cooked sample. In comparison, RedBu_1_, RedBa_1_, WhiteLi_1_, RedBu_2_, RedBa_2_ and WhiteLi_2_ exhibited the greatest antioxidant activity at the digested and fermented stages of processing. However, these varieties exhibited a decrease in activity by approximately 50% for digested samples when fermentation with subsequent cooking was implemented.

**Figure 2:**
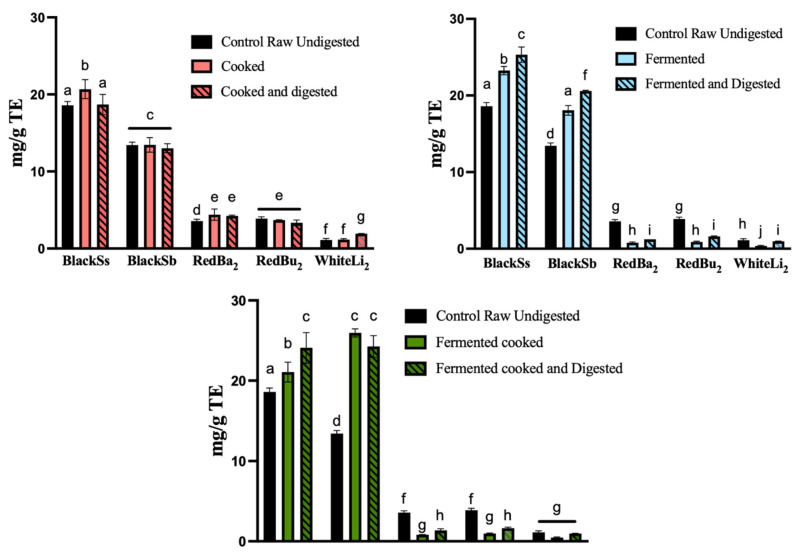
Differences in the total FRAP radical scavenging activity of processed sorghum phenolic extracts before and after simulated gastrointestinal digestion. Data are expressed as mg/g TE, and represent mean ± SD; *n =* 3. Significant differences within and between sorghum samples are represented by different letters. Horizontal lines indicate no significant difference within sorghum samples. WhiteLi_2_, Liberty from Croppa Creek; Bazley from Croppa Creek; RedBu_2_, Buster from Croppa Creek; BlackSs, Shawaya short black 1; BlackSb, Shawaya black.

The radical scavenging activity using FRAP of five processed sorghum samples was significantly increased by digestion (*p* < 0.05), which correlates with the ABTS results shown in Figure 1. Digested BlackSs and BlackSb showed an increase in activity following the fermented and fermented-cooked processes (Figure 2). Prior to digestion, BlackSs exhibited the highest activity (18.59 ± 0.49 mg/g TE). The radical scavenging activity of digested BlackSs increased for all processes, with 90% retained after digestion of the cooked samples. Interestingly, the digested FRAP activity of BlackSs was elevated by 14% and 9% after the fermented and fermented-cooked processes were implemented, respectively. Conversely, BlackSb exhibited the second highest activity at 13.44 ± 0.37 mg/g TE. This value increased by approximately 3-fold after the fermented-cooked process, and a further 7% post-digestion (Figure 2). Similar to the black varieties, the digested RedBa_2_, RedBu_2_ and WhiteLi_2_ varieties showed an increase in activity when samples were fermented or fermented-cooked, but WhiteLi_2_ did not demonstrate a significant difference pre- and post-digestion.

### 2.3. Influence of Different Growing Locations on the TPC of Processed and Digested Sorghum

The TPC of the digested red and white sorghum samples was significantly influenced by the growing location post-processing (*p* < 0.0001) (Figure S1). Among the three varieties investigated (Liberty, Bazley and Buster), the growing location of Bellata, NSW exhibited significantly higher levels in total phenolic content compared to Croppa Creek, NSW (*p* < 0.05). Specifically, the phenolic content of RedBa_1_ increased from 0.47 ± 0.12 to 0.72 ± 0.11 mg/g GAE post-processing, with a further increase to 1.00 ± 0.13 mg/g GAE post-digestion. RedBu_1_ also increased by approximately 28% post-processing, and 100% of this content was retained after digestion. WhiteLi_1_ showed a slight but significant decrease in phenolic content, but interestingly digestion increased the phenolic content by 2-fold (0.45 ± 0.05 to 0.82 ± 0.07 mg/g GAE). This was at the same level for processed and digested WhiteLi_2_.

### 2.4. UHPLC—Online ABTS and QTOF LC-MS Identification and Quantification of Phenolic Compounds Post In Vitro Digestion and Caco-2 Intestinal Transport of Processed Sorghum

Table 2 lists 58 peaks corresponding to compounds identified in the UHPLC-QTOF LC-MS profiles of processed and undigested, processed and digested and Caco-2 transported (processed and digested) sorghum samples. Out of the 34 compounds identified prior to digestion, more than 50% were detected after in vitro digestion, including phenolic acids, flavonol glucosides, flavan-3-ols and stilbenes (Table 3, Figure S2). Eight compounds were detected after Caco-2 intestinal transport (Table 3). These compounds were procyanidin a1 (**5**), glutamine (**6**), kaempferol-3-*O*-glucuronide (**8**), petunidin-3-(6-*O*-coumaroyl) glucoside (**16**), tryptophan (**19**), epicatechin gallate (**28**), catechin (**33**) and 2,4,6-trihydroxyphenanthrene-2-*O*-glucoside (**53**). These polyphenols were identified via the comparison of chromatographic data with analytical standards, online databases and the existing literature.

**Figure 3:**
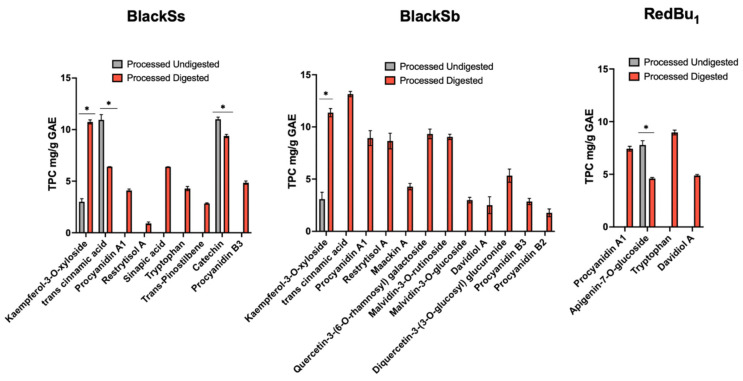
Recovery of phenolic compounds from BlackSs, BlackSb and RedBu_1_ post in vitro digestion (determined by UHPLC–Online ABTS and gallic acid standard curve). Data are expressed as mg/g GAE and presented as mean ± standard deviation (*n =* 3). * Statistically significant difference between total phenolic content (TPC) and retrieval of phenolic compounds following in vitro digestion. RedBu_1_; Buster from Bellata; BlackSs, Shawaya short black 1; BlackSb, Shawaya black.

The UHPLC–Online ABTS quantification of sorghum polyphenols detected a greater number of compounds in the black samples compared to the red and white ones. The phenolic profile of BlackSs was significantly altered following digestion. Out of the 21 identified compounds pre-digestion (Figure S3), 9 were recovered after in vitro digestion (Figure 3 and Figure S2). The flavonol glycoside, kaempferol-3-*O*-xyloside (**1**), exhibited a significant 3-fold increase in TPC post-digestion, from 3.01 ± 0.29 to 10.74 ± 0.19 mg/g GAE. Conversely, trans-cinnamic acid (**12**) decreased by approximately 50% post-digestion, similar to catechin (**33**), which decreased from 11.00 ± 0.20 to 9.38 ± 0.16 mg/g GAE. Several compounds emerged following in vitro digestion, including procyanidin a1 (**5**), restrytisol a (**9**), sinapic acid (**13**), tryptophan (**19**), trans-pinostilbene (**21**) and procyanidin b3 (**40**). For BlackSb, which initially contained 12 compounds, a total of 12 were also recovered after in vitro digestion (Figure 3 and Figure S4). The phenolic acid, trans-cinnamic acid (**12**), was the predominant compound detected post-digestion at 13.15 ± 0.25 mg/g GAE; however, this compound was not detected pre-digestion. Similar to BlackSs, the compound kaempferol-3-*O*-xyloside (**1**) increased in TPC from 3.09 ± 0.65 to 11.37 ± 0.40 mg/g GAE. Three isomers of procyanidin, two isomers of quercetin and two isomers of malvidin, which were previously not identified in the undigested phase, were recovered post-digestion. Interestingly, the stilbenes restrytisol a (**9**), maackin a (**15**) and davidiol a (**29**) were also recovered post-digestion of BlackSb (Figure 3). RedBu_1_ initially contained 20 compounds, with a total of 4 compounds recovered following in vitro digestion. The flavone glycoside, apigenin-7-*O*-glucoside (**7**), significantly decreased from 7.79 ± 0.40 to 4.60 ± 0.11 mg/g GAE. Tryptophan (19) was the predominant compound in RedBu_1_, followed by procyanidin a1 (**5**) and davidiol a (**29**); these compounds also appeared after in vitro digestion. Generally, the phenolic content increased following digestion, correlating with the benchtop TPC results shown in Table 1.

### 2.5. Transport of Digested Sorghum Phenolic Compounds Through the Basolateral Chamber of the In Vitro Caco-2 Intestinal Monolayer

The absorption of digested sorghum polyphenols (bioavailability) was studied using an in vitro monolayer of Caco-2 cells. The percentage of transported compounds was determined by comparing their peak areas at 280 nm with those in the digested samples of BlackSs, BlackSb and RedBu_1_ in the UHPLC–Online ABTS coupled QTOF LC-MS system. The outcomes demonstrate that 100% of the glutamine (**6**), kaempferol-3-*O*-glucuronide (**8**), epicatechin gallate (**28**) and 2,4,6-Trihydroxyphenanthrene-2-*O*-glucoside (**53**) were transported through the basolateral Caco-2 intestinal monolayer (Table 3). Petunidin-3-(6-*O*-coumaroyl) glucoside (**16**) was only detected in BlackSb and catechin (**33**) was only detected in BlackSs, both at a rate of 100%. Interestingly, procyanidin a1 (**5**) exhibited reduced transport at 6% for BlackSs and 6% for RedBu_1_. In contrast, the same phenolic compound was retrieved from the basolateral chamber at a rate of 22% for BlackSb. Several new peaks were identified in the Caco-2 transported sorghum samples that were not detected following simulated digestion, explaining the elevated retrieval percentages shown in Table 3.

## 3. Materials and Methods

### 3.1. Materials

#### 3.1.1. Sorghum Samples

Black, red and white sorghum wholegrains (BlackSb, BlackSs, RedBa_1_, RedBu_1_, WhiteLi_1_, RedBa_2_, RedBu_2_, and WhiteLi_2_) were acquired as described previously [6]. Briefly, the red and white samples were sourced from the 2021 field trials conducted in Bellata and Croppa Creek in New South Wales. Black wholegrain sorghum was sourced from the 2021 glasshouse trials conducted in Warwick, Queensland. The sorghum samples were maintained at 4 °C prior to experimentation. Triplicate experimental analyses were performed for each sample.

#### 3.1.2. Chemicals, Standards and Reagents

Folin-Ciocalteu reagent, Iron (III) chloride, formic acid, TPTZ, ABTS, Trolox, acetic acid, acetone, hydrochloric acid, hexane, methanol, potassium persulfate, sodium carbonate and sulfuric acid were acquired from Sigma-Aldrich (St. Louis, MO, USA) and Chem Supply Pty Ltd. (Port Adelaide, South Australia, Australia). The simulated digestion assay was conducted as described by Ed Nignpense et al. (2022) [18]. Briefly, the chemical HCl, pepsin and pancreatin enzymes and bile extract were acquired from Sigma-Aldrich. The standards for phenolic identification included apigenin, caffeic acid, catechin, chlorogenic acid, coumarin, cyanidin-3-*O* glucoside, delphinidin chloride, ellagic acid, ferulic acid, gallic acid, hippuric acid, isovanillic acid, luteolin, naringenin, o-coumaric acid, p-coumaric acid, peonidin 3-*O* glucoside chloride, petunidin 3-*O* glucoside, phloridzin, phosphotungstic acid, procyanidin B1, procyanidin B3, protochatechuic acid, quercetin, rutin, sinapic acid, syringic acid, trans-cinnamic acid, vanillin and vanillic acid were purchased from Sigma-Aldrich. The cell culture materials included Dulbecco’s modified eagle’s medium (DMEM), Eagle’s minimum essential medium (EMEM), fetal bovine serum (FBS) and 6-well transmembrane plates (Corning, CLS3412), all purchased from Sigma-Aldrich. Trypsin and penicillin–streptomycin were sourced from Thermo Fisher Scientific (Waltham, MA, USA), and the Caco-2 colorectal cancer cells were purchased from the American Type Culture Collection (Manassas, VA, USA).

### 3.2. Methods

**Figure 4:**
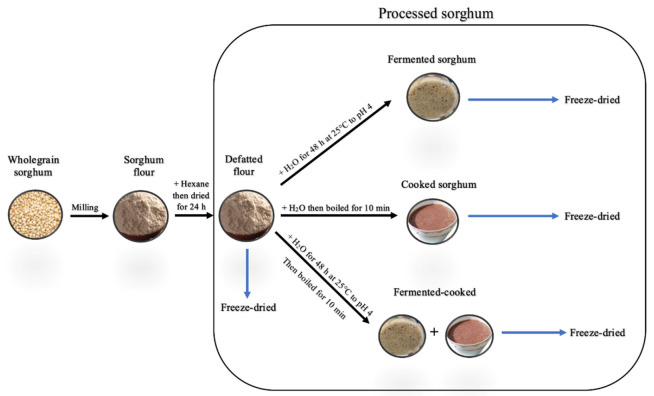
Sorghum preparation flow chart depicting how fermented, cooked and fermented-cooked samples are obtained.

Processed sorghum wholegrains were prepared as previously described by Collins et al., 2024b [11]. Briefly, sorghum flour was procured by milling wholegrain sorghum into a flour (Figure 4). The flour was defatted using hexane three times, then left to dry in a fume cabinet for 24 h. The flour fermentation process was adapted from Correia et al. (2010) [19], with minor modifications. Briefly, a mixture of sorghum flour and deionised water (10 g in 20 mL) was left to ferment at room temperature (25 °C) for 48 h to pH 4 before adding 80 mL of deionised water to stop fermentation. Sorghum porridge was cooked by adding a slurry of previously fermented or unfermented sorghum flour (10 g in 20 mL) to 80 mL of boiling water, stirring for 10 min then cooling in a water bath [20]. The processed sorghum samples were freeze-dried and stored at 4 °C for further analysis. Samples preparation was performed in triplicates.

### 3.3. Simulated Digestion of Processed Sorghum Using an In Vitro Approach

The in vitro simulated digestion assay by Ed Nignpense et al. (2022) [18] was used with minor modifications. Briefly, 3 g of cooked, fermented or fermented-cooked sorghum flour were homogenized in a solution of saline then sonicated for 30 min at 25 °C. This mixture was then acidified to pH 2 with 5 mL of 0.1 M HCl and 0.2 g pepsin enzyme. The solution was incubated for 1 h at 37 °C to initiate gastric digestion. To mimic the intestinal digestion phases, the gastric digesta pH was changed to 6.9 by adding sodium carbonate (NaHCO_3_). Pancreatic bile (0.075 g pancreatin and 0.45 g bile extract in 37.5 mL of 0.1 M NaHCO_3_) was added while the mixture was incubated for 2 h at 37 °C in order for digestion to occur. The resulting digesta was centrifuged at 4000 rpm for 10 min. The supernatants were retrieved and freeze-dried, and a concentration of 1 g/mL in 50% methanol was used for further analysis. A digestion blank devoid of sorghum samples was used to account for any background interference from reagents and enzymes. The sample supernatant was used for complete polyphenol evaluation using chromatography and mass spectrometry.

### 3.4. Determination of Phenolic Content and Antioxidant Potential

#### 3.4.1. Total Phenolic Content (TPC)

Determination of TPC was implemented as described by Rao et al. (2018) [21] with slight variations. A 125 μL aliquot of digested sorghum and 125 μL of Folin-Ciocalteu reagent were mixed with a volume of 500 μL sterile water then incubated for 6 min in the dark. A volume of 1.5 mL of 7% Na_2_CO_3_ was added along with 1 mL of sterile water to stop the reaction. After 90 min in the dark at room temperature, a microplate reader (BMG Labtech Fluostar Omega, Offenburg, Germany) was used to measure the absorbance at 760 nm. A standard curve of gallic acid was generated to quantify TPC of the samples, and the data were expressed as mg/g Gallic acid equivalents (GAE). Each replicate was repeated three times for the analyses.

#### 3.4.2. Radical Scavenging Activity Using the ABTS Assay

The antioxidant ABTS activity of the sample was assessed as described by Rao et al. (2018) [21]. In brief, 50 µL of the sample was added to 1 mL of ABTS reagent and allowed to react at room temperature for 30 min. The absorbance was then recorded at 734 nm using a spectrophotometer, and the ABTS activity was reported as mg of Trolox equivalents per 100 g (mg 100 g^−1^ TE). A standard curve of Trolox was utilised and results were displayed as mg 100 g^−1^ TE. Each measurement was performed in triplicate.

#### 3.4.3. Antioxidant Activity Using the Ferric Reducing Antioxidant Potential (FRAP) Assay

The FRAP benchtop assay was adapted from Sompong et al. (2011) [22] following slight changes. FRAP reagent comprised of 100 mL acetate buffer, 10 mL FeCl_3_.6H_2_O and 10 mL TPTZ solution in a ratio of 10:1:1 was freshly prepared. A volume of 1.8 mL aliquot was mixed with 60 μL of digested sorghum sample and 180 μL of deionised water and vortexed. Following a 40 min incubation at 37 °C, a microplate reader was used to read the absorbance at 593 nm. Trolox was used to generate a standard curve and determine the antioxidant activity (measured in mg/g TE). Each experimental replicate was performed in triplicate.

### 3.5. UHPLC—Online ABTS Analysis of Sorghum Samples

Polyphenol characterization was performed following the method described by Collins et al. (2024b) [11]. Briefly, aliquots of 20 μL reconstituted sorghum samples were inoculated into the Agilent UHPLC system (Agilent Technologies, Santa Clara, CA, USA) with a 2.1 mm × 50 mm, 1.8 µm Zorbac Eclipse Plus C18 column. The system comprised a coil column, exterior binary pump and UV-vis detector that injected 0.6 mL ABTS solution per minute. Mobile phase A contained 0.01% formic acid in deionized water, while Mobile phase B contained 0.01% formic acid in acetonitrile. The elution gradient was 34.98 min consisting of 0–50% A and B, 34.98–37.21 min with 100% B and 37.31–39.65 min with 100% B. Compound peaks were measured at an absorbance of 280 nm and 414 nm for their respective antioxidant activity. A Trolox equivalent (TE) standard curve was generated to determine the ABTS radical scavenging activity (mg 100 g^−1^ TE). The peaks were detected according to retention time and chromatogram spectra and quantified against standards as mg/g GAE.

### 3.6. QTOF LC-MS Sorghum Phenolic Compound Identification

The compound peaks were identified as described by Collins et al. (2024b) [11]. The peaks were identified using negative mode, and a comprehensive *m*/*z* scan was performed from 50 to 1500. Qualitative Analysis software using Agilent Mass Hunter version B.07.00 was used to extract QTOF chromatograms, and the unknown peaks were determined using standards and the online literature.

### 3.7. Caco-2 Cellular Transport Assay

The cellular transport assay using Caco-2 colon adenocarcinoma cells was conducted as described by Hilary et al. (2020) [15] with slight modifications. Caco-2 cells were added to in 6-well transmembrane plates at a density of 0.3 × 10^6^ cells per insert (4.67 cm^2^, 0.4 μm pore size). Using DMEM supplemented with FBS and penicillin–streptomycin (89%, 10% and 1% respectively), the cells were cultured for approximately 15 days at 37 °C in an incubator set at 5% CO_2_. The basolateral chamber of the plate received DMEM devoid of Caco-2 cells. The cell culture medium was replenished every 2 to 3 days until a cell monolayer was formed. The formation and integrity of the monolayer were determined by measuring the TEER using a MilliCell voltammeter (Millicell ERS-2, Merck Millipore, Billerica, MA, USA). A TEER charge above 400 Ω/cm^2^ confirmed the formation and integrity of the monolayer. The digested sorghum samples were diluted with DMEM (1:1 vol/vol), then added onto the Caco-2 apical layer. Incubation was performed for 4 h at 37 °C in a 5% CO_2_ incubator to replicate human gastrointestinal digestion conditions. At the end of the 4 h incubation, the apical and basolateral culture media were collected and lyophilised. The subsequent samples were reconstituted with 1 mL of sterile water, filtered through a 0.22-μm filter then analysed for polyphenol composition as per Section 2.5.

### 3.8. Statistical Analysis

One-way analysis of variance (ANOVA) was conducted using GraphPad Prism 9 software (GraphPad Software Inc., San Diego, CA, USA). Post hoc comparisons were determined using Tukey’s test, and the results are presented as mean ± standard deviation. Statistical significance was completed at *p* < 0.05.

## 4. Discussion

The aim of this study was to assess how simulated gastrointestinal digestion affects the phenolic composition and antioxidant activity of processed sorghum samples (WhiteLi_1_; RedBa_1_; RedBu_1_; WhiteLi_2_; RedBa_2_; RedBu_2_; BlackSs and BlackSb). Our previous study indicated that these samples exhibit increased polyphenol accessibility due to the degradation of polyphenols and the cell-wall matrix resulting from fermentation and the heat treatment during cooking [11]. In order to attain the health effects of processed sorghum polyphenols in vivo, the compounds must withstand digestion and become available for absorption in the gastrointestinal tract. The current study provides the first report on bioaccessible polyphenols in sorghum that are efficiently absorbed and transported across intestinal cells; as demonstrated using an in vitro approach.

Our findings revealed that the TPC as determined by Folin–Ciocalteu significantly increased following digestion for all samples (Table 1). The increase in TPC of processed sorghum after digestion can be attributed to the breakdown of complex phenolic compounds into smaller, more bioavailable forms [23]. During digestion, enzymatic hydrolysis and acidic conditions in the gastrointestinal tract can convert bound phenolic compounds into free phenolic acids, which possess higher antioxidant activity [24]. The higher TPC observed for digested RedBa_1_ and RedBu_1_ grown in Bellata, NSW compared to those grown in Croppa Creek, NSW, can be attributed to several environmental and agronomic factors specific to this region (Figure S1). A warmer climate may improve the synthesis of secondary metabolites, including phenolic compounds that are more bioaccessible, as plants often produce these compounds as a defence mechanism against environmental stressors such as UV radiation and drought [25]. Additionally, the region’s soil may contain specific micronutrients or organic matter that stimulate the production of phenolic compounds in wholegrain sorghum [26]. Overall, the interplay of climatic conditions, soil properties and agricultural practices in Bellata likely contributes to the elevated total phenolic content observed in red sorghum from this area.

The antioxidant activity of processed sorghum as determined by ABTS and FRAP assays also increased post-digestion, with BlackSs exhibiting the most pronounced increase (Figure 2 and Figure 3). Interestingly, the ABTS antioxidant activity of the digested red and white samples decreased when fully processed (fermented and cooked). We hypothesize that due to the scarcity of compounds resistant to enzymatic digestion (e.g., proanthocyanidins), the degradation of phenolic compounds can occur in the digestive medium [27]. Conversely, digested BlackSs and BlackSb displayed increasing activities for each process, demonstrating that these samples are abundant in compounds not susceptible to chemical and enzymatic digestion conditions. Xiong et al. (2019) [28] have similarly reported the unique flavonoid 3DXA, which is present in darker pigmented sorghum varieties and resistant to structural breakdown as a result of a missing hydroxyl group at the carbon-3 ring. The surge in antioxidant activity correlates with the total phenolic content results, suggesting that digestion causes alterations in the features of phenolic compound structures, thereby raising their antioxidant capacity. A study by Adarkwah-Yiadom and Duodu (2017) [14] found that sorghum extrusion cooking progressively increased the total phenolic and tannin content, which is consistent with our findings. Moreover, the transformation of polyphenols into simpler structures can result in an increase in their bioavailability and, consequently, their antioxidant efficacy. This bio-conversion process reflects a dynamic interaction between dietary processing, enzymatic action and microbial fermentation, contributing to the observed elevation in antioxidant properties of digested sorghum [29].

The in vitro digestion of processed BlackSs, BlackSb and RedBu_1_ revealed bioaccessible compounds that have not previously been reported in sorghum, including trans-pinostilbene (**21**), tryptophan (**19**), maackin a (**15**), restrytisol a (**9**) and davidiol a (**29**). BlackSb exhibited the greatest recovery of compounds post-digestion, with a total of twelve compounds detected (Figure 3). The only polyphenol detected pre- and post-digestion of BlackSb was kaempferol-3-*O*-xyloside (**1**). This compound significantly increased by 4-fold in TPC, likely due to the stability in the hydroxyl group of this flavonol glycoside. Conversely, three compounds were detected before and after simulated digestion of BlackSs, including kaempferol-3-*O*-xyloside (**1**), trans-cinnamic acid (**12**) and catechin (**33**); however, the latter two compounds decreased in TPC by 42% and 15%, respectively. However, this decrease seemingly did not significantly affect the overall phenolic content of BlackSs. These results are similar to investigations by Adelakun et al. (2017) [2] and Salazar-López et al. (2018) [20], who reported an increase in total phenolics and antioxidant activity following the digestion of thermally processed sorghum. Although BlackSs exhibited a higher recovery of polyphenols after digestion, a total of nine compounds were detected post Caco-2 transport. The flavonoid glycoside, apigenin-7-*O*-glucoside [7], was unique to RedBu_1_ due to its absence in the other samples. This compound may have strong covalent bonds capable of withstanding the pH conditions of the simulated digestion assay [28].

Transport experiments using Caco-2 cells showed that flavone glycosides, flavan-3-ols and phenolic acids were identified as the main transported class of polyphenols, as described in Table 2. There were variations in the absorption levels among compounds, including glutamine (**6**), kaempferol-3-*O*-glucuronide (**8**) and epicatechin gallate (**28**), across the sorghum samples. Procyanidin a1 (**5**) showed lower transport efficacy for BlackSs and RedBu_1_ (6%) than for BlackSb (22%). These findings suggest that the same compound in different pigmented sorghum varieties may have altered solubility or permeability properties, influencing its ability to cross the cellular monolayer. Furthermore, tryptophan (**19**) was detected at higher transport rates in BlackSb and BlackSs (83% and 100%) than in RedBu_1_ (45%). Notably, the metabolites glutamine (**6**), kaempferol-3-*O*-glucuronide (**8**), 2,4,6-trihydroxyphenanthrene-2-*O*-glucoside (**53**) and epicatechin gallate (**28**) were detected in the basolateral layer for all sorghum samples, but were not present in the apical layer (Table 3, Figure S2). This compound was likely converted from epigallocatechin when an ester bond is formed between the galloyl and hydroxyl group, thereby producing the bioavailable form, epicatechin gallate (**28**). Additionally, petunidin-3-(6-*O*-coumaroyl) glucoside (**16**) and catechin (**33**) were only detected in BlackSb and BlackSs, respectively. A possible explanation for the emergence of these different compounds could be due to metabolic processes within the Caco-2 cells or interactions with endogenous enzymes (**16**). Investigations by Bergonzi et al. (2023) [30] determined that when a compound with a glucose segment is introduced to the apical layer of the Caco-2 intestinal cells, it may be subject to enzymatic cleavage by cellular glycosidases. These enzymes hydrolyse the glycosidic bond, releasing the glucose bond and yielding the parent compound. The released compound can then permeate the epithelial barrier and appear on the basolateral side, reflecting its bioavailability. Similarly, in our study, sorghum polyphenols were also detected on the basolateral side following Caco-2 transport, suggesting that these compounds, after potential enzymatic modification, were successfully absorbed through the epithelial barrier.

The Caco-2 assay, as a model of the human intestinal epithelium, provides valuable insights into the bioavailability and absorption of compounds, even though it may not replicate all aspects of human digestion and metabolism [15]. The variation in Caco-2 transported sorghum polyphenols can also be attributed to their chemical structure, solubility and the transport mechanisms that regulate their movement [3]. This includes passive diffusion for simple phenolic acids and flavonoid aglycones, versus facilitated transport via glucose transporters for flavonoid glycosides [31]. Importantly, a single phenolic acid was transported across the monolayer, whereas several flavonoid glycosides and flavan-3-ols were detected after intestinal absorption (Table 2), underscoring the need for further research to elucidate the precise cellular transport mechanisms of these compounds and their implications for enhancing bioavailability.

Sorghum processing methods, such as cooking and fermentation, significantly influence the physiochemical properties of phenolic compounds, which can affect their absorption and bioavailability [11]. While the Caco-2 assay presents certain limitations, such as the lack of enteroendocrine cells and a complex in vivo environment, it is important to acknowledge why our findings regarding the increased TPC and antioxidant activity of digested and processed sorghum polyphenols remain scientifically relevant. Firstly, our research demonstrates a marked increase in TPC and antioxidant activity post-digestion, which is consistent with biochemical transformations observed in similar studies [3,15]. The in vitro simulated digestion process used in our study mimics the conditions of the gastrointestinal tract, including enzymatic and acidic environments, which facilitate the breakdown of complex polyphenolic structures into more bioavailable forms. This is supported by our results showing that polyphenols, previously bound within the sorghum matrix, are released and converted into more active phenolic compounds following digestion.

The observed increase in antioxidant activity and TPC can be associated with the liberation of bound polyphenols and their subsequent absorption across the Caco-2 monolayer. This finding is aligned with the well-documented ability of digestive processes to enhance the bioavailability of polyphenols through enzymatic cleavage and microbial fermentation [32]. Furthermore, while the Caco-2 model may not capture the full complexity of the human gut microbiota or the entire digestive process, it provides a controlled environment to assess fundamental interactions between digested polyphenols and intestinal cells. The increase in antioxidant activity observed in our assay reflects the enhanced bioavailability and potential biological activity of the polyphenols, which are supported by similar results in other in vitro and in vivo studies. Consequently, the metabolic fate and bioactivity of compounds observed in vitro may differ from those observed in vivo, impacting the translatability of findings to human health outcomes. Hence, while simulated digestion models offer valuable insights into initial digestion and nutrient bioaccessibility, complementary human dietary intervention studies using sorghum grain polyphenols remain essential for validating these findings and elucidating comprehensive physiological responses to dietary components.

## 5. Conclusions

This study underlines the complex interactions between digestive processes, phenolic composition, and antioxidant activity in processed sorghum. We determined that in vitro digestion increases the bioaccessibility of processed sorghum polyphenols as a result of elevated antioxidant activity and total phenolic content across the eight varieties evaluated. The Caco-2 intestinal transport assay indicated that flavone glycosides, flavan-3-ols and phenolic acids from processed sorghum were likely detected following enzymatic activity changes and cellular biotransformation mechanisms. Furthermore, the crop’s growing location showed an impact on the level of antioxidant activity and phenolic content, including compounds that were highly bioaccessible. The findings in this study suggest that processed sorghum polyphenols can potentially reach the colon for microbial modifications for notable attributable health benefits in the gastrointestinal tract. Further investigations into the specific mechanisms governing these interactions using human intervention studies are warranted to elucidate the potential applications of the bioaccessible compounds identified in this study.

## Figures and Tables

**Table 1:** Total phenolic content as affected by digestion following sorghum processing.

**TPC (mg/g GAE)**								
Sorghum Process	BlackSs	BlackSb	RedBu_1_	RedBa_1_	WhiteLi_1_	RedBu_2_	RedBa_2_	WhiteLi_2_
R	5.05 ± 1.18 a	6.05 ± 1.82 a	0.67 ± 0.08 a	0.47 ± 0.14 a	0.52 ± 0.06 a	0.58 ± 0.07 a	0.53 ± 0.08 a	0.52 ± 0.05 a
C	10.00 ± 1.14 b	10.51 ± 1.91 b	0.69 ± 0.11 a	0.52 ± 0.10 a	0.49 ± 0.05 a	0.69 ± 0.14 a	0.68 ± 0.07 b	0.62 ± 0.21 ab
C DIG	9.20 ± 0.81 bc	10.05 ± 1.71 bc	0.96 ± 0.10 b	0.94 ± 0.14 b	0.91 ± 0.09 b	0.91 ± 0.11 b	0.89 ± 0.13 c	0.89 ± 0.13 b
F	6.55 ± 0.55 d	6.90 ± 1.84 a	0.84 ± 0.06 c	0.73 ± 0.12 c	0.65 ± 0.07 c	0.65 ± 0.11 c	0.71 ± 0.12 b	0.79 ± 0.17 c
F DIG	9.25 ± 1.70 be	9.71 ± 1.37 bd	1.03 ± 0.08 bd	1.02 ± 0.13 bd	0.95 ± 0.09 bd	0.88 ± 0.11 bd	0.97 ± 0.11 ce	0.85 ± 0.11 bcd
FC	6.79 ± 1.21 df	6.58 ± 1.15 a	0.93 ± 0.07 e	0.72 ± 0.11 ce	0.45 ± 0.05 a	0.58 ± 0.07 ae	0.62 ± 0.11 b	0.64 ± 0.18 ae
FC DIG	9.94 ± 1.88 bg	9.30 ± 1.87 bde	0.93 ± 0.11 ef	1.00 ± 0.13 bf	0.82 ± 0.07 e	0.83 ± 0.13 f	0.83 ± 0.12 g	0.83 ± 0.09 e

Data are expressed as mg/g GAE, and represent mean ± SD; *n =* 9. Significant differences within and between sorghum samples are represented by different letters. The letters bc, be, bg and bf etc. indicate a significant difference with fermented or fermented-cooked samples but no significant difference with cooked samples. R, raw; C, cooked; F, fermented; FC, fermented and cooked; DIG, digested; WhiteLi_1_, Liberty from Bellata; WhiteLi_2_, Liberty from Croppa Creek; RedBa_1_, Bazley from Bellata; RedBa_2_, Bazley from Croppa Creek; RedBu_1_, Buster from Bellata; RedBu_2_, Buster from Croppa Creek; BlackSs, Shawaya short black 1; BlackSb, Shawaya black.

**Table 2:** Identification of sorghum phenolic peaks after processing, simulated digestion and in vitro Caco-2 intestinal transport using QTOF LC-MS.

Peak	Tentative ID	*m*/*z*	RT	Polyphenol Class
1 **°**	Kaempferol-3-*O*-xyloside	418.9579	0.80	Flavonol glycoside
2	Trans-Piceid	251.0797	1.06	Stilbene
3 **°**	Trans-cinnamic acid	147.0310	1.07	Phenolic acid
4	6-Methoxy-7-hydroxycoumarin	193.0360	1.09	o-hydroxycinnamic acid
5 **°^∆^**	Procyanidin A1	539.1378	1.22	Flavan-3-ol
6 **^∆^**	Glutamine	128.0360	1.64	Digestion metabolite
7 **°**	Apigenin-7-*O*-glucoside	413.1686	2.16	Flavone glycoside
8 **^∆^**	Kaempferol-3-*O*-glucuronide	267.0744	2.20	Flavone glycoside
9 **°**	Restrytisol A	377.0864	2.30	Stilbene
10	Coumarin	145.0510	2.47	o-hydroxycinnamic acid
11	Protocatechuic acid	153.0198	3.00	Phenolic acid
12 **°**	Trans-cinnamic acid	147.0445	3.09	Phenolic acid
13 **°**	Sinapic acid	164.0718	3.20	Phenolic acid
14 **°**	Apigenin-7-*O*-glucoside	413.1679	3.65	Flavone glycoside
15 **°**	Maackin A	485.2382	3.84	Stilbene
16 **^∆^**	Petunidin-3-(6-*O*-coumaroyl) glucoside	218.1028	4.13	Flavone glycoside
17	Catechin	289.0726	5.14	Flavan-3-ol
18 **°**	Quercetin-3-(6-*O*-rhamnosyl) galactoside	540.2797	5.45	Flavone glycoside
19 **°^∆^**	Tryptophan	203.0819	6.20	Phenolic amino acid
20	4-Acetylbutyric acid	131.0721	7.55	Phenolic acid
21 **°**	Trans-Pinostilbene	241.1204	7.74	Stilbene
22	Caffeic acid	181.0512	7.75	Phenolic acid
23	Procyanidin B1 isomer	577.1386	8.97	Flavan-3-ol
24	Catechin	289.0733	9.08	Flavan-3-ol
25 **°**	Malvidin-3-*O*-rutinoside	330.2048	9.12	Flavone glycoside
26	2-Isopropylmalic acid	177.0201	9.20	Phenolic acid
27 **°**	Malvidin-3-*O*-glucoside	512.2386	9.57	Flavone glycoside
28 **^∆^**	Epicatechin gallate	440.1326	10.05	Flavan-3-ol
29 **°**	Davidiol A	567.3513	10.30	Stilbene trimer
30	Apigeninidin	253.0737	10.43	3-deoxyanthocyanidin
31	Procyanidin C1	865.1978	10.45	Flavan-3-ol
32	Epigallocatechin	307.1409	11.42	Flavan-3-ol
33 **°^∆^**	Catechin	289.0739	11.46	Flavan-3-ol
34	Trans-cinnamic acid	147.0453	11.79	Phenolic acid
35	Isoferulic acid	195.0668	12.09	Phenolic acid
36 **°**	Diquercetin-3-(3-*O*-glucosyl) glucuronide	756.3681	12.13	Flavone glycoside
37	Catechin derivative	720.1572	12.73	Flavan-3-ol
38	N′.n′-dicafferoylspermidine	468.2130	13.21	Phenolic acid
39	Ferulic acid	468.2156	13.56	Phenolic acid
40 **°**	Procyanidin B3	579.3146	14.05	Flavan-3-ol
41	Malic acid	135.0458	14.53	Phenolic acid
42	Catechin	289.0738	14.60	Flavan-3-ol
43 **°**	Procyanidin B2	451.2577	14.61	Flavan-3-ol
44	Glucomalcomiin	482.2334	15.39	o-hydroxycinnamic acid
45	Kaempferol	187.0980	15.67	Flavonol
46	Epicat-(4beta→6)-epicatechin-(2beta→7,4beta→8)-epicatechin	867.2377	16.36	Flavan-3-ol
47	Procyanidin C1	867.2382	16.37	Flavan-3-ol
48	Luteolin-7-*O*-glucoside	447.0954	16.81	Flavone glycoside
49	Pyrano-eriodictyol-(3→4)-catechin-7-*O*-glucoside	867.2362	16.99	Flavone glycoside
50	Taxifolin	303.0527	17.69	Flavononol
51	Procyanidin	429.2133	17.98	Flavan-3-ol
52	Pyrano-naringenin-(3→4)-catechin-7-*O*-glucoside isomer	851.2415	18.55	Flavone glycoside
53 **^∆^**	2,4,6-Trihydroxyphenanthrene-2-*O*-glucoside	353.0496	18.63	Flavone glycoside
54	Taxifolin	303.0879	19.23	Flavanonol
55	Pentahydroxyflavanone-(3→4)-catechin-7-*O*-glucoside isomer	721.1780	19.99	Flavone glycoside
56	Luteolin	285.0766	21.45	Flavone
57	7-*O*-methyl-luteolinidin	395.2135	21.49	3-deoxyanthocyanidin
58	Luteolin derivative	415.1070	22.41	Flavone

The symbol **°** indicates that the compound was detected after the in vitro digestion phases. The symbol **^∆^** indicates the compound was detected after Caco-2 intestinal monolayer transport. The symbols **°^∆^** indicate that the compound was detected after the in vitro digestion phase and Caco-2 intestinal monolayer transport.

**Table 3:** Transport of compounds from processed and digested sorghum samples through the Caco-2 monolayer.

Compound	% BlackSs Compound Retrieval	% BlackSb Compound Retrieval	% RedBu_1_ Compound Retrieval
5	6 ± 1 a	22 ± 2 b	6 ± 0 a
6	100 ± 2 c	100 ± 1 c	100 ± 5 c
8	100 ± 6 c	100 ± 21 c	100 ± 3 c
16	-	100 ± 33 c	-
19	83 ± 6 d	100 ± 1 c	45 ± 1 e
28	100 ± 2 c	100 ± 23 c	100 ± 1 c
33	100 ± 11 c	-	-
53	100 ± 3 c	100 ± 3 c	100 ± 12 c

Data are presented as mean ± standard deviation (*n =* 3). Different letters indicate significant differences within and between sorghum samples. RedBu_1_, Buster from Bellata; BlackSs, Shawaya short black 1; BlackSb, Shawaya black. The percentage bioaccessibility of polyphenols was calculated by dividing the concentration of digested compounds in the apical layer by the transported compounds in the basolateral chamber and multiplying by 100. ANOVA was performed with multiple comparisons, and statistical significance was set at *p* ≤ 0.05.

## Data Availability

The original contributions presented in the study are included in the article, further inquiries can be directed to the corresponding authors.

## Supplementary Materials

The following supporting information can be downloaded at: https://www.mdpi.com/article/10.3390/molecules29225229/s1, Figure S1: The total phenolic content of processed and digested sorghum as influenced by different growing locations, Figure S2: UHPLC—Online ABTS and QTOF LC-MS identification of phenolic compounds in undigested, digested and Caco-2 transported processed sorghum. Figure S3. Undigested (processed) BlackSs sorghum extract UHPLC-Online ABTS profile at 280 nm and 414 nm. Figure S4. UHPLC chromatogram of BlackSb before digestion (grey), after digestion (red) and after in vitro Caco-2 cellular transport (green).
