# Supplementary material for: Effect of In Vitro Gastrointestinal Digestion on the Polyphenol Bioaccessibility and Bioavailability of Processed Sorghum (Sorghum bicolor L. Moench)
Source: Molecules. 2024 Nov 5;29(22):5229. doi: 10.3390/molecules29225229 (PMC11596331; doi:10.3390/molecules29225229)
Supplement: Supplementary file 1 [file molecules-29-05229-s001.zip › Figure S1.pdf]

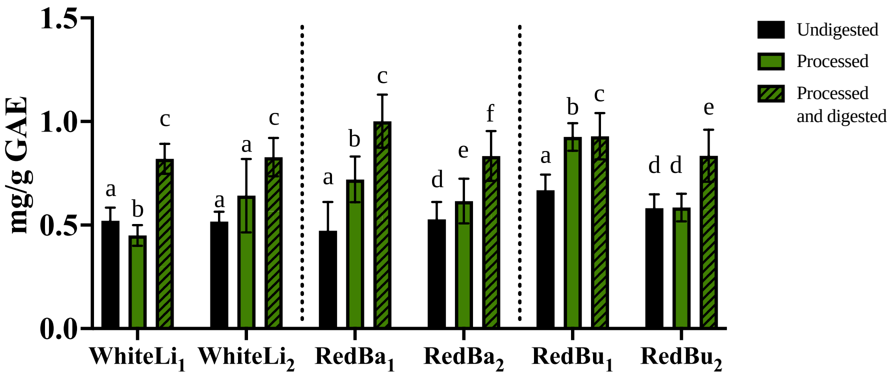

Figure S1. Variations in the total phenolic content (TPC) of the same sorghum varieties from different growing locations post processing (fermentation and cooking) and digestion using the Folin-Ciocalteu assay. Data are blank corrected, presented as mg/g GAE and expressed as means ± SD; n = 9. Level of significance is indicated by different letter using a two-way ANOVA with Fisher's LSD test. $WhiteLi_1$, Liberty from Bellata; $WhiteLi_2$, Liberty from Croppa Creek; $RedBa_1$; Bazley from Bellata, $RedBa_2$; Bazley from Croppa Creek, $RedBu_1$; Buster from Bellata, $RedBu_2$, Buster from Croppa Creek; BlackSs, Shawaya short black 1; BlackSb, Shawaya black.
