# Supplementary material for: Effect of In Vitro Gastrointestinal Digestion on the Polyphenol Bioaccessibility and Bioavailability of Processed Sorghum (Sorghum bicolor L. Moench)
Source: Molecules. 2024 Nov 5;29(22):5229. doi: 10.3390/molecules29225229 (PMC11596331; doi:10.3390/molecules29225229)
Supplement: Supplementary file 1 [file molecules-29-05229-s001.zip › Figure S2.pdf]

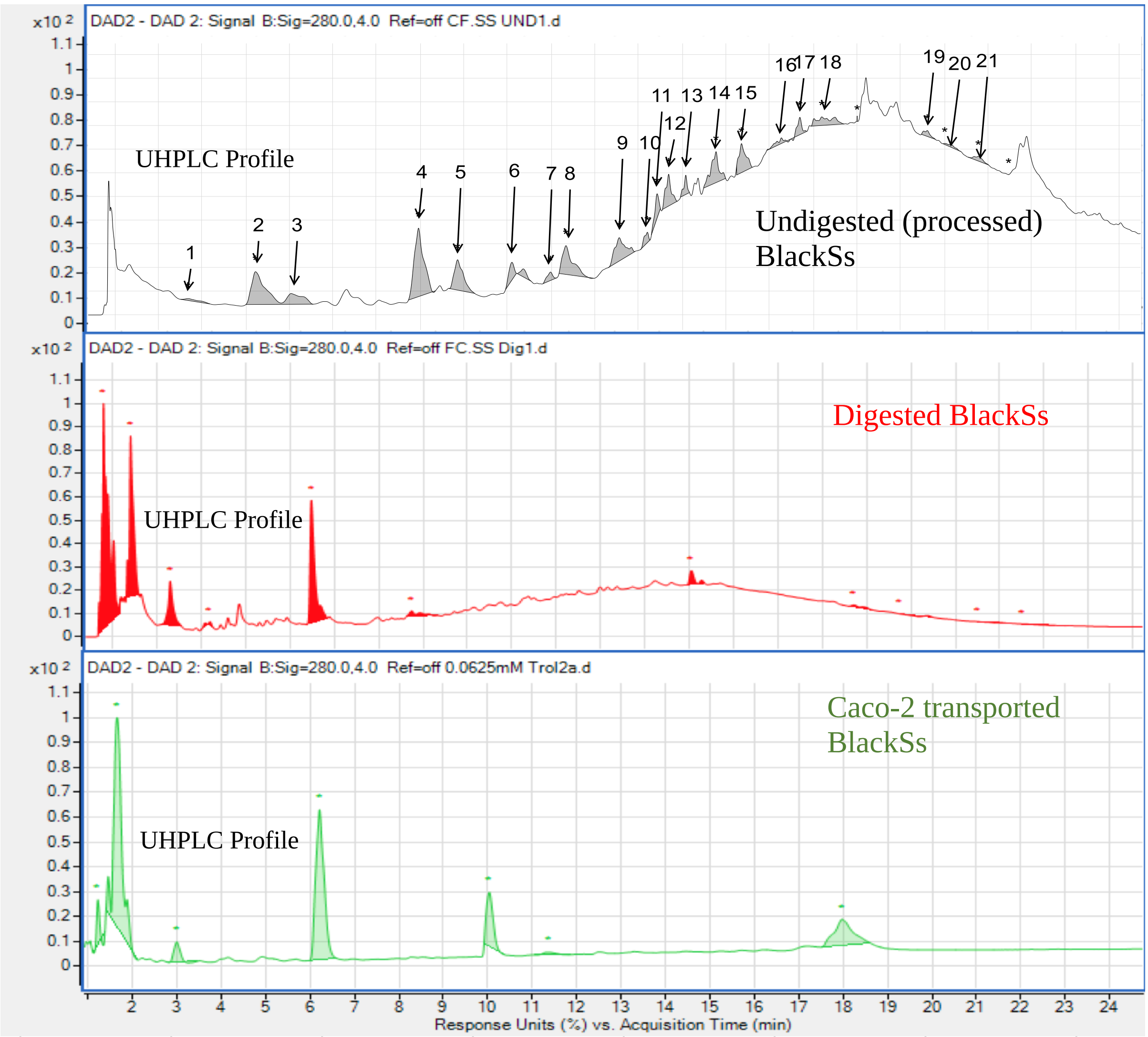

Figure S2. UHPLC chromatogram of BlackSs before digestion (grey), after digestion (red) and after *in vitro* Caco-2 cellular transport (green). The symbol * indicated that the compound was detected in multiple processes.
