# Supplementary figures and images for: Effect of In Vitro Gastrointestinal Digestion on the Polyphenol Bioaccessibility and Bioavailability of Processed Sorghum (Sorghum bicolor L. Moench)
Source: Molecules. 2024 Nov 5;29(22):5229. doi: 10.3390/molecules29225229 (PMC11596331; doi:10.3390/molecules29225229)

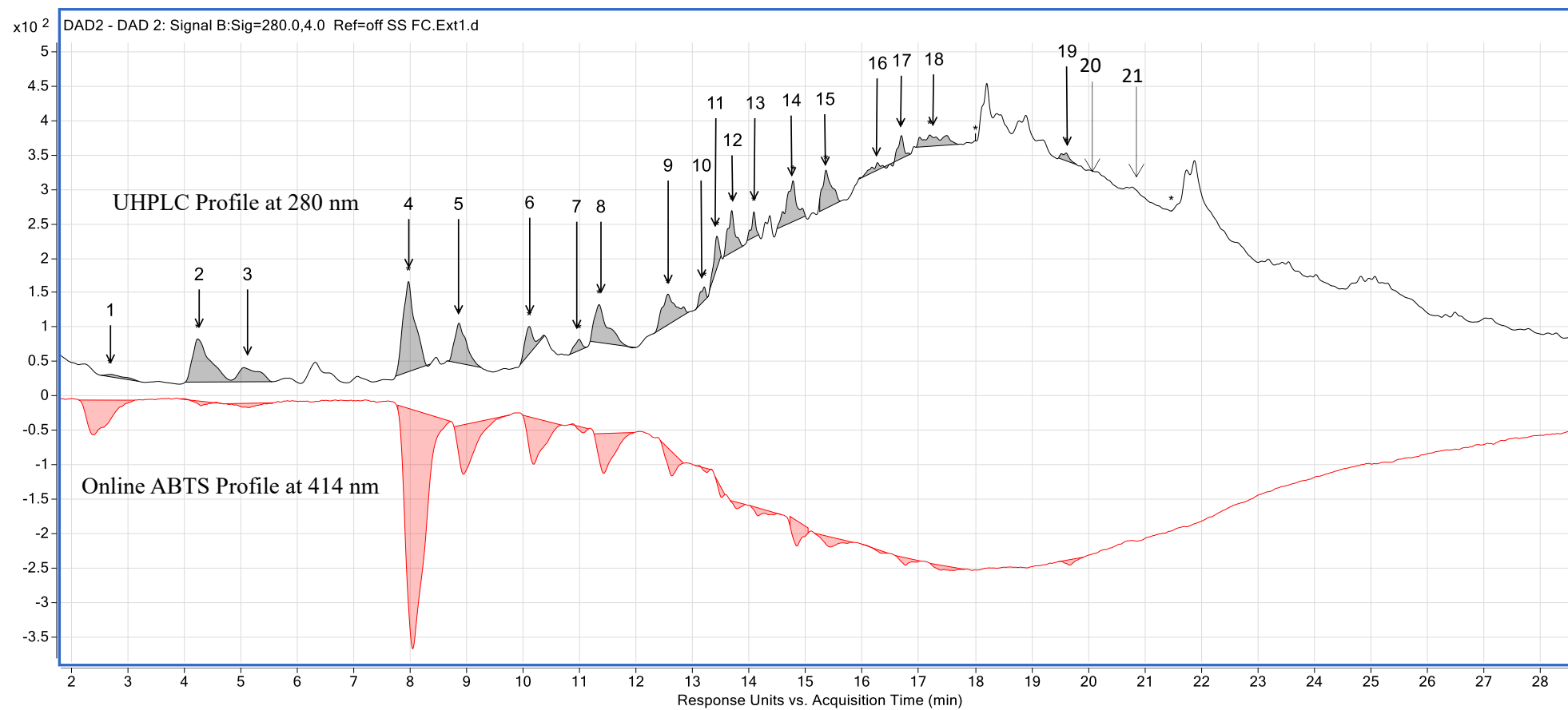

**Figure S3.** Undigested (processed) BlackSs sorghum extract UHPLC-Online ABTS profile at 280 nm and 414 nm.

Supplement: Supplementary file 1 [file molecules-29-05229-s001.zip › Figure S3.pdf]
